# Centennial Review: A meta-analysis of the significance of *Eimeria* infection on apparent ileal amino acid digestibility in broiler chickens^1^

**DOI:** 10.1016/j.psj.2021.101625

**Published:** 2021-12-01

**Authors:** Emily Kim, Marie-Pierre Létourneau-Montminy, William Lambert, Tristan Chalvon-Demersay, Elijah G. Kiarie

**Affiliations:** ⁎ Department of Animal Biosciences, University of Guelph, Guelph, ON, Canada N1G 2W1; † Département des Sciences Animales, Université Laval, Québec city, QC, Canada; ‡ METEX NOOVISTAGO, Paris, France

**Keywords:** broiler chickens, amino acids digestibility, meta-analysis, *Eimeria* challenge

## Abstract

*Eimeria* infections impair digestive tract capacity and barrier function leading to poor growth and feed efficiency. A meta-analysis approach was used to evaluate and quantify impact of *Eimeria* infection on the apparent ileal digestibility (**AID**) of amino acids (**AA**) in broiler chickens. A database composed of 6 articles with a total of 21 experiments was built for the effect of challenge type (a mix of *Eimeria* spp. vs. *E. acervulina*) and subdatabase of 3 articles with a total of 15 experiments for the effect of *E. acervulina* dose response. Regression models were fitted with the mixed model procedure in Minitab 19 with fixed effects of challenge, species, and their interactions. For the sub database, the mixed model procedure was used to fit regression models and identify a linear or quadratic response to dose. Challenge decreased AID (*P* < 0.05) of both dispensable and indispensable AA except for Trp. Specifically, the largest depression was observed for Cys, Thr, Tyr, Ala, and Val with the magnitude of difference of 8.7, 5.4, 5.2, 5.1, and 4.9%, respectively for challenged vs. unchallenged birds. The type of challenge affected (*P* < 0.05) AID of AA with exception of Cys, Tyr, Ala, Ser, Leu, Asp, Gly, and Pro. *E. acervulina* challenge had larger negative effects on AID of Ile, Leu, and Val. Moreover, *E. acervulina* linearly decreased (*P* < 0.0*5*) AID of all indispensable and dispensable AA except for Trp and quadratically (*P* < 0.05) decreased AID of all AA except Cys, Met, Arg, and Trp. The largest linear decrease due to *E. acervulina* dose was seen for AID of Cys, followed by Ala, Val, Thr, and Ile. Although, AID of Trp was not affected by *E. acervulina* challenge, mixed *Eimeria* species challenge decreased (*P* < 0.05) AID of Trp*.* Overall, the results confirmed that an *Eimeria* infection negatively impacted AA digestibility/utilization. The ranking of the most affected AA suggested ground for nutritional intervention during subclinical field *Eimeria* infections or vaccination programs.

## INTRODUCTION

Avian coccidiosis is a parasitic disease caused by the apicomplexan protozoa *Eimeria* that results in significant damage to the host intestinal cells, therefore incurring considerable economic losses for poultry producers (Chapman, 2009, 2014). Traditionally, in-feed anticoccidial compounds have been used as the prevention method of choice, however, drug-resistant *Eimeria* species and consumer concern over drug residues in poultry products have challenged the industry to move away from the chemotherapeutic control of coccidiosis and move toward nonmedicated methods such as vaccination (Lee et al., 2011; Price, 2012). Live oocyst vaccines, using either attenuated or nonattenuated *Eimeria,* have been shown to be quite successful in stimulating protective immunity through the exposure of *Eimeria* antigens (Williams, 2002). Despite their effectiveness in providing protection against coccidiosis, the major disadvantage to utilizing these vaccines is the age at which they are administered to the chicks, often within the first week of life (Chapman, 2014). At a time crucial for small intestinal development, an enteric disturbance provoked by the vaccination not only increases a chick susceptibility to secondary infections such as necrotic enteritis, but may evoke an early reduction in growth and later impact body weight gain and feed efficiency (Chapman et al., 2005; Chapman, 2014). As such, the reluctance of broiler producers to embrace vaccination programs is correlated to several reports where the performance between vaccinated and medicated birds has not been equal (Williams, 2002; Lee et al., 2011). However, moving in favor of drug-free poultry products means that without the use of anticoccidial drugs, broiler chickens must rely on their own immune system to fight off clinical or subclinical disease (Klasing, 2007). Thus, nutrients such as amino acids (**AA**) that would have otherwise been allocated for growth and other metabolic processes, are redirected toward sustaining the immune system (Korver, 2012).

Currently, broiler diets are formulated in accordance with the digestible and ideal AA concepts, thus there exists a ratio of AA that should provide the exact balance of AA needed for optimum performance and growth (Dalibard et al., 2014; Rochell et al., 2016b). However, this ratio may change during a broiler lifespan due to age or physiological state (Dalibard et al., 2014). In this manner, coccidiosis may alter the ideal AA profile due to biochemical and physiological changes caused by disease pathogenesis. For example, an increase in mucin production, typically associated with an *Eimeria* infection, may increase the demand for Thr, Gly, and Ser, therefore increasing endogenous AA requirement (Lehman et al., 2009). This suggests that the AA requirement of broilers raised in drug-free programs may differ from broilers raised in conventional practices and begs the question of whether there are essential, conditionally or even nonessential AA that may become limiting when broilers, raised in drug-free programs in particular, are subjected to enteric disturbances. Therefore, a deeper understanding of the influence of an *Eimeria* challenge on AA digestibility in broilers is needed to help identify potential limiting AA that can minimize the detriments of an *Eimeria* infection.

There are numerous studies that have evaluated the impact of *Eimeria* infection models on AA digestibility in broiler chickens (e.g., Adedokun et al. 2012, 2016; Rochell et al., 2016a,b,c; Teng et al., 2021), however, studies often focus on specific questions and its difficulty to integrate data to propose recommendations. To our knowledge, there is a lack of studies focused on quantifying the effect of an *Eimeria* challenge on the apparent ileal digestibility (**AID**) of AA, much less the standardized ileal digestibility of AA in broilers. The use statistical method methods such as meta-analysis may aid in summarizing and quantifying knowledge from the results of published research studies (Sauvant et al., 2008). Thus, the objective of the present study was to use a meta-analysis approach to evaluate and quantify the impact of *Eimeria* infection on the AID of AA in broiler chickens.

## MATERIALS AND METHODS

### Database Collection and Coding

An article search was performed in Google Scholar and in the Omni database to retrieve articles based on the inclusion of key words [broilers, *Eimeria* challenge, and AA digestibility] in their titles. The articles retrieved were then included based on the following criteria: 1) an *Eimeria* challenge with at least 1 control and 1 challenged group, 2) broilers of any age, 3) nutritional composition of the diet available, and 4) measurements of ileal AA digestibility values. Articles were excluded if: 1) studies were not conducted in broilers, 2) studies only reported apparent total tract AA digestibility values, and 3) studies were not published in a peer-reviewed journal. After an exhaustive search, the database compiled 5 articles (Table 1) published between 2016 and 2020. This database included general information (i.e., journal of publication, author name), qualitative data (i.e., breed, sex, *Eimeria* species), and quantitative data (i.e., nutritional feed composition, ileal AA digestibility). This overall dataset yielded a total of 13 experiments and was built to look at the effect of challenge and type of challenge (*E. acervulina* challenge *vs*. a mix of *Eimeria* spp.) on ileal AA digestibility. Due to the limited number of studies reporting standardized ileal digestibility values, apparent ileal digestibility (**AID**) was chosen as the dependent variable. Additionally, there was interest to see the effect of dose response to *Eimeria* on AID of AA. As such, a subdatabase made up of 2 of the 5 articles that tested different doses of *Eimeria* with a total of 7 experiments that was used to evaluate the dose effect on AID of AA of *E. acervulina*-challenged birds. A code was assigned to each publication and each experiment in the databases to categorize the data.

**Table 1:** List of comparison studies used in meta-analysis.

Publication	Objective	Bird breed	Challenge code^1^	Challenge type	Concentration of oocysts^3^	Challenge duration^4^
1	*Eimeria* dose effect	Ross 308	0	*E. acervulina*	0.00	6
1	*Eimeria* dose effect	Ross 308	1	*E. acervulina*	2.50	6
1	*Eimeria* dose effect	Ross 308	1	*E. acervulina*	5.00	6
1	*Eimeria* dose effect	Ross 308	1	*E. acervulina*	10.00	6
2	Age	Ross 708	0	mix^2^	0.00	6
2	Age	Ross 708	1	mix	0.06	6
3	Cu supplementation and AA level	Ross 308	0	*E. acervulina*	0.00	15
3	Cu supplementation and AA level	Ross 308	1	*E. acervulina*	6.33	15
4	Diet type and vaccination type	Cobb 500	0	mix	0.00	12
4	Diet type and vaccination type	Cobb 500	1	mix	0.0489	12
5	*Eimeria* dose effect	Ross 308	0	mix	0.00	10
5	*Eimeria* dose effect	Ross 308	1	mix	0.0383	10

Publications: Adedokun et al. (2016), Rochell et al. (2016a), Rochell et al. (2016b), Gautier and Rochell (2020).

1 Challenge code (0 = not challenged, 1 = challenged).

2 Challenge type mix species consist of *E. acervulina, E. maxima, E. mivati, E. tenella.*

3 Concentration of oocysts (1.0 × 10^5^/mL).

4 Measured in days.

### Data Examination and Statistical Analyses

The potential variables and interactions evaluated in the meta-analyses were tested as predictors of AA digestibility using Minitab 19 software (State College, PA). This included but was not limited, to the effect of challenge, challenge type, challenge duration, age of inoculation, breed, diet, and concentration of oocysts. The significant variables were then retained in the models. Descriptive statistics (mean, standard deviation, and range of values) for each variable is shown in Table 2. Preliminary scatterplots of the models (not shown) were created to allow a visual representation of the relationship between the dependent variable and independent variable, as well as recognition of noticeable outliers. In addition, all studies were verified for crude protein content (%), apparent metabolizable energy (kcal/kg), and SID Lys requirement (mg/day) in accordance with the nutrient specification guidelines of the corresponding breed and age during the challenge period to avoid any confounding effects on AA digestibility from inadequate nutrient provision.

**Table 2:** Descriptive statistics of databases.

	AID									
Database	Overall^1^					*Acervulina*^2^				
No. of experiments	13					7				
No. of treatments	26					14				
Independent variable	n	Mean	SD	Min	Max	n	Mean	SD	Min	Max
Challenge	26					14				
Challenge type	26					14				
Oocyst concentration	26	1.66	2.97	0.00	10.00	14	3.06	3.51	0.00	10.00
Dependent variable										
Digestibility, %										
Arg	26	86.73	3.84	71.50	90.80	14	88.23	2.54	82.40	90.80
His	26	81.80	5.56	58.80	86.50	14	83.92	2.88	77.30	86.50
Ile	26	79.73	5.77	57.10	85.20	14	81.55	4.16	72.90	85.20
Leu	26	81.68	5.15	63.10	87.20	14	83.40	4.22	74.30	87.20
Lys	26	83.79	3.52	73.00	88.30	14	85.26	3.13	78.40	88.30
Met	26	89.39	3.08	81.10	93.60	14	90.51	3.09	85.40	93.60
Phe	26	80.78	6.38	56.00	87.30	14	83.42	4.00	75.10	87.30
Thr	26	73.73	6.31	49.50	79.80	14	76.01	4.16	67.10	79.80
Trp	20	82.28	10.96	56.90	90.10	14	88.81	0.93	87.10	90.10
Val	26	77.93	6.13	53.80	84.00	14	80.12	4.32	71.10	84.00
Ala	26	79.84	5.97	57.60	86.00	14	81.70	4.53	72.20	86.00
Asp	26	77.69	5.37	56.90	82.90	14	79.29	3.65	71.30	82.90
Cys	26	66.34	8.42	43.40	75.40	14	66.24	8.46	44.10	73.20
Glu	26	84.90	4.55	68.40	89.40	14	86.41	3.31	79.20	89.40
Gly	26	74.83	5.36	53.80	80.00	14	75.56	3.51	68.20	80.00
Pro	26	80.06	5.52	58.10	85.00	14	81.54	3.93	72.60	85.00
Ser	26	77.34	5.69	55.10	83.00	14	79.06	3.86	70.90	83.00
Tyr	16	82.67	3.12	76.20	86.60	14	82.98	3.13	76.20	86.60

Abbreviations: Ala, alanine; Asp, aspartic acid; Arg, arginine; Cys, cysteine; Glu, glutamic acid; Gly, glycine; His, histidine; Ile, isoleucine; Leu, leucine; Lys, lysine; Met, methionine; n, number of studies; Phe, phenylalanine; Pro, proline; Ser, serine; Thr, threonine; Trp, tryptophan; Tyr, tyrosine; Val, valine.

Publications: Adedokun et al. (2016), Rochell et al. (2016a), Rochell et al. (2016b), Gautier and Rochell (2020).

1 Overall database contained all studies.

2 *Acervulina* database contained studies with just *E. acervulina* challenge.

For the overall database, regression models were fitted with the MIXED procedure in Minitab 19 with fixed effects of challenge, the challenge type, and their interactions. For the *acervulina* database, the MIXED model procedure was used to fit regression models and identify a linear-quadratic response to dose. Subsequently, the fit of each relationship was examined by the studentized residuals of the model and normal probability plots of residuals to identify and remove outliers accordingly. The experiment code was considered as a random effect as the databases consisted of multiple different studies in which each study contributes a random outcome from a collection of studies (St-Pierre, 2001; Sauvant et al., 2008). Two sample *t* tests were performed to compare the difference between 2 slopes of predictions of AA digestibility. The averaged *P*-value for each AA was then used to create a hierarchization of which AA was most affected to least affected. An alpha level of 0.05 was used as the criterion for statistical significance.

## RESULTS

The models were evaluated separately for each of the datasets. The effects of diet, challenge duration, age of inoculation, and breed were initially investigated, however, were not significant and therefore not included in the final models. Additionally, experiment was considered as a random effect, however, was not significant and therefore dropped from the model.

### Overall Database Models

**Figure 1:**
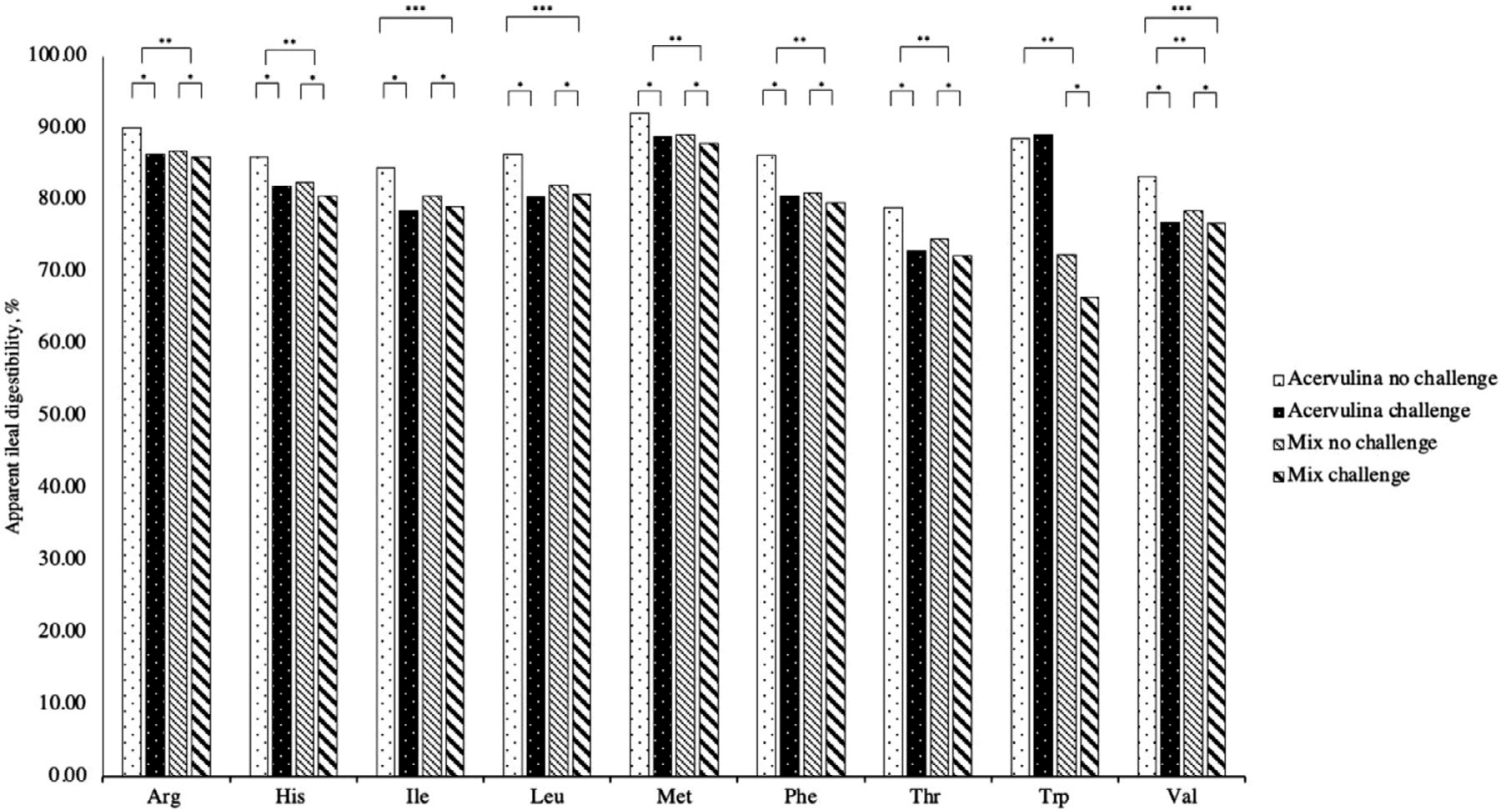
Impact of challenge and challenge type on apparent ileal amino acid digestibility of indispensable amino acids. Abbreviations: Arg, arginine; His, histidine; Ile, isoleucine; Leu, leucine; Lys, lysine; Met, methionine; Phe, phenylalanine; Thr, threonine; Trp, tryptophan; Val, valine. * = effect of challenge, *P* < 0.05. ** = effect of challenge type (*E. acervulina* vs. mix), *P* < 0.05. *** = effect of challenge and challenge type interaction, *P* < 0.05*.*

**Figure 2:**
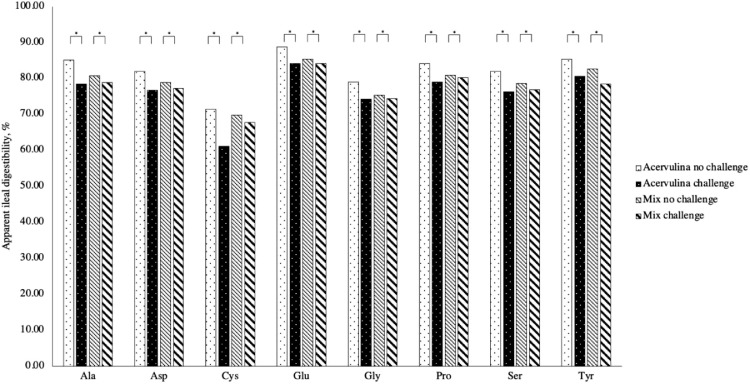
Impact of challenge and challenge type on apparent ileal amino acid digestibility of dispensable amino acids. Abbreviations: Ala, alanine, Asp, aspartic acid; Cys, cysteine; Glu, glutamic acid; Gly, glycine; Pro, proline; Ser, serine; Tyr, tyrosine. * = effect of challenge, *P* < 0.05. ** = effect of challenge type (*E. acervulina* vs. mix), *P* < 0.05. *** = effect of challenge and challenge type interaction, *P* < 0.05*.*

The general regression equations for measuring AID of AA from the overall database had an intercept different from 0 (*P* < 0.05; Table 3). For the impact on AA digestibility, the challenge effect (coefficient A) was significant for Arg, His, Ile, Leu, Lys, Phe, Thr, Val, Ala, Asp, Ser (*P <* 0.01), Met, Cys, Glu, Gly, Pro, Tyr (*P* < 0.05), such that digestibility decreased with challenge (Figure 1, Figure 2). The largest effects were seen in Cys, Thr, Tyr, Ala, Val, Ile, and Ser (8.7, 5.3, 5.2, 5.1, 4.9, 4.6, and 4.5% magnitude of difference with challenge, respectively). Conversely, challenge effect was not significantly related to Trp digestibility (*P* = 0.136). The challenge type effect (coefficient B) was significant (*P* < 0.05) for most indispensable AA except for Ile and Leu, such that digestibility values of the mixed *Eimeria* spp. challenges were lower than (*P* < 0.05) that of the *E. acervulina* challenges, however, challenge type was not significantly related to dispensable AA*.* The interaction between challenge and challenge type (coefficient C) was not significant for most AA except for Ile, Leu, and Val, where digestibility decreased significantly with the *E. acervulina* challenge compared to the mixed *Eimeria* challenge. The *t* test comparing the model slopes of challenge ranked digestibility of Cys most affected by challenge, followed by Arg, Met, Ala, Tyr, and Thr (Table 4).

**Table 3:** Impact of challenge and challenge type on apparent ileal amino acid digestibility.

	AID						
		*P*-value					
Amino acid	Regression equation	Intercept	Challenge	Challenge type	Challenge × Challenge type	RMSE	R2, %
Arg	y=87.30+1.11a+0.90b+0.66c	<0.001	0.003	0.013	0.060	1.60	55.89
His	y=82.70+1.49a+1.20b+0.53c	<0.001	0.004	0.017	0.264	2.22	50.41
Ile	y=80.70+1.89a+0.84b+1.09c	<0.001	0.002	0.117	0.046	2.49	54.39
Leu	y=82.42+1.78a+0.98b+1.17c	<0.001	0.004	0.087	0.044	2.63	51.73
Lys	y=84.06+1.56a+1.20b+0.72c	<0.001	0.001	0.007	0.085	1.92	60.56
Met	y=89.52+1.10a+0.99b+0.54c	<0.001	0.027	0.045	0.255	2.24	38.94
Phe	y=81.86+1.7a+1.56b+1.05c	<0.001	0.003	0.008	0.061	2.56	57.00
Thr	y=74.73+2.07a+1.27b+0.90c	<0.001	0.000	0.049	0.155	2.95	50.18
Trp	y=79.14+1.36a+9.66b-1.65c	<0.001	0.136	0.000	0.075	3.02	90.37
Val	y=78.89+2.01a+1.22b+1.12c	<0.001	0.001	0.031	0.045	2.54	58.23
Ala	y=80.75+2.12a+0.95b+1.19c	<0.001	0.002	0.134	0.064	2.94	51.43
Asp	y=78.65+1.69a+0.63b+0.90c	<0.001	0.004	0.240	0.100	2.52	46.41
Cys	y=67.48+3.07a-1.24b+2.03c	<0.001	0.031	0.358	0.140	6.38	33.58
Glu	y=85.55+1.45a+0.86b+0.85c	<0.001	0.012	0.117	0.120	2.54	42.33
Gly	y=75.72+1.44a+0.85b+0.93c	<0.001	0.017	0.139	0.107	2.66	40.86
Pro	y=81.03+1.45a+0.51b+1.07c	<0.001	0.017	0.371	0.067	2.68	40.22
Ser	y=78.37+1.83a+0.69b+1.00c	<0.001	0.002	0.192	0.066	2.49	51.30
Tyr	y=81.76+2.19a+1.22b+0.14c	<0.001	0.016	0.143	0.857	2.06	65.16

Abbreviations: Ala, alanine; Asp, aspartic acid; Arg, arginine; Cys, cysteine; Glu, glutamic acid; Gly, glycine; His, histidine; Ile, isoleucine; Leu, leucine; Lys, lysine; Met, methionine; Phe, phenylalanine; Pro, proline; Ser, serine; Thr, threonine; Trp, tryptophan; Tyr, tyrosine; Val, valine.

y = apparent ileal amino acid digestibility (%).

a = effect of challenge (1 = not challenged, -1 = challenged).

b = effect of challenge type (1 = *acervulina,* -1 = mix).

c = effect of challenge × challenge type (1 = *acervulina* not challenged, mix challenged, -1 = *acervulina* challenged, mix not challenged.

**Table 4:** Ranking of amino acid digestibility affected by challenge.

Amino acid	Rank^1^
Cys	1
Arg	2
Met	3
Ala	4
Tyr	5
Thr	6
Val	7
Gly	8
Lys	9
Ile	10
His	11
Glu	12
Pro	13
Ser	14
Phe	15
Asp	16
Trp	17
Leu	18

Abbreviations: Ala, alanine; Asp, aspartic acid; Arg, arginine; Cys, cysteine; Glu, glutamic acid; Gly, glycine; His, histidine; Ile, isoleucine; Leu, leucine; Lys, lysine; Met, methionine; Phe, phenylalanine; Pro, proline; Ser, serine; Thr, threonine; Trp, tryptophan; Tyr, tyrosine; Val, valine.

1 Ranking from most affected by challenge to least affected.

### *Acervulina* Database Models

**Figure 3:**
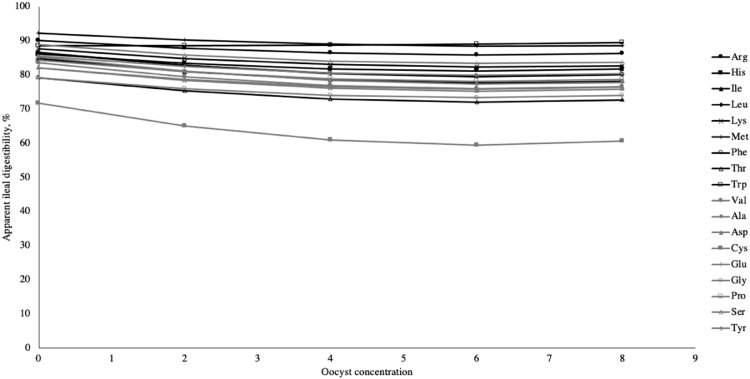
Oocyst concentration^1^ effect on apparent ileal amino acid digestibility. Abbreviations: Ala, alanine; Arg, arginine; Asp, aspartic acid; Cys, cysteine; Glu, glutamic acid; Gly, glycine; His, histidine; Ile, isoleucine; Leu, leucine; Lys, lysine; Met, methionine; Phe, phenylalanine; Pro, proline; Ser, serine; Thr, threonine; Trp, tryptophan; Tyr, tyrosine; Val, valine. ^1^ oocyst concentration (1.0 × 10^5^/mL).

The general regression equations for measuring AID of AA tested from the *Acervulina* database had an intercept different from 0 (*P* < 0.05; Table 5). For the impact on AA digestibility, *E. acervulina* dose of oocyst (coefficient X) linearly decreased for all AA (*P* < 0.0*1*) except for Trp (*P* = 0.822) (Figure 3). Oocyst concentration also quadratically decreased digestibility (*P* < 0.05) for all AA except for Arg, Met, Trp, Cys, Glu, and Pro.

**Table 5:** Impact of oocyst concentration on apparent ileal amino acid digestibility.

	AID					
		*P*-value				
Amino acid	Regression equation	Intercept	Linear	Quadratic	RMSE	R2, %
Arg	y=90.14-1.38x+0.11x^2^	0.000	0.007	0.051	1.29	83.29
His	y=86.09-1.61x+0.13x^2^	0.000	0.005	0.035	1.36	86.50
Ile	y=84.75-2.29x+0.18x^2^	0.000	0.004	0.035	1.88	87.64
Leu	y=86.56-2.25x+0.18x^2^	0.000	0.006	0.046	1.93	87.78
Lys	y=87.68-1.69x+0.13x^2^	0.000	0.003	0.035	1.31	89.85
Met	y=92.24-1.19x+0.09x^2^	0.000	0.032	0.155	1.37	90.72
Phe	y=86.44-2.16x+0.17x^2^	0.000	0.005	0.042	1.83	87.57
Thr	y=79.17-2.30x+0.19x^2^	0.000	0.003	0.030	1.80	89.22
Trp	y=88.54-0.03x+0.02x^2^	0.000	0.822	0.333	0.39	92.46
Val	y=83.48-2.42x+0.19x^2^	0.000	0.003	0.031	1.95	87.44
Ala	y=85.23-2.46x+0.19x^2^	0.000	0.004	0.037	1.99	88.31
Asp	y=82.08-2.04x+0.16x^2^	0.000	0.004	0.035	1.71	86.39
Cys	y=71.69-4.00x+0.33x^2^	0.000	0.019	0.096	4.23	86.58
Glu	y=88.89-1.78x+0.14x^2^	0.000	0.006	0.050	1.60	85.82
Gly	y=79.14-1.94x+0.16x^2^	0.000	0.008	0.049	1.86	81.93
Pro	y=84.27-2.02x+0.16x^2^	0.000	0.010	0.062	1.90	86.90
Ser	y=82.09-2.21x+0.18x^2^	0.000	0.002	0.022	1.65	88.99
Tyr	y=85.48-1.80x+0.14x^2^	0.000	0.003	0.027	1.47	85.00

Abbreviations: Ala, alanine; Asp, aspartic acid; Arg, arginine; Cys, cysteine; Glu, glutamic acid; Gly, glycine; His, histidine; Ile, isoleucine; Leu, leucine; Lys, lysine; Met, methionine; Phe, phenylalanine; Pro, proline; Ser, serine; Thr, threonine; Trp, tryptophan; Tyr, tyrosine; Val, valine

y = apparent ileal amino acid digestibility (%).

x = oocyst concentration (1.0 × 10^5^/mL).

## DISCUSSION

The current study was conducted to better understand the impact of an *Eimeria* challenge on apparent ileal digestibility of AA. There are seven species of *Eimeria,* the protozoan parasite and causative agent for avian coccidiosis, that have been recognized to be pathogenic along different regions of the intestinal tract (Chapman, 2014). *E. acervulina* and *E. praecox* typically reside in the duodenum, which can extend to mid-intestine in cases of heavy infections, *E. mitis, E. maxima,* and *E. necatrix* tend to develop in the mid- to posterior small intestine, while *E. tenella* and *E. brunetti* are found in the ceca and rectum, respectively (Chapman, 2014). The *Eimeria* invade and proliferate within gut epithelial cells, causing damage and compromising digestion and absorption of nutrients in the small intestine, subsequently increasing ileal endogenous AA secretions and reducing AA digestibility (Adedokun et al., 2012; Chapman, 2014; Kiarie et al., 2019). However, protein synthesis is an inevitable and necessary process in the animal that requires a certain supply of essential and nonessential AA to be present at the site of synthesis, depending on the requirements of the animal (Dalibard et al., 2014). Thus, when an essential AA is limited, protein synthesis is also limited (Dalibard et al., 2014). Hence, a sufficient quantity of limiting AA governs the efficiency of AA utilization (Dalibard et al., 2014). In the case of coccidiosis, many studies have shown higher AA requirements for the performance and well-being of broilers. In a study by Murillo et al. (1976), the growth performance of *Eimeria-*infected broilers was more depressed when fed a Met + Cys-deficient diet compared to broilers fed a Met + Cys-sufficient diet. Moreover, broilers fed the Met + Cys-sufficient diet had increased levels of jejunum luminal immunoglobulin (**Ig**) A, an important antibody that plays a role in the immune function of mucosal membranes (Murillo et al., 1976). In another study by Corzo et al. (2007), broilers were fed the same diet and either reared on new litter or recycled litter. The group raised on recycled litter had a higher requirement for Thr compared to the new litter group. The authors hypothesized that an increased use of Thr for mucin synthesis as a result of inflammation in the gut may have explained their results (Corzo et al., 2007). Therefore, certain AA may become limiting when the health status of the bird is threatened and understanding which AA are most affected may be a key element in mitigating the consequences of an *Eimeria* infection on growth performance.

In the current study, multiple linear regression models were fitted to evaluate and quantify the impact of an *Eimeria* challenge on AID of AA, specifically focusing on the effect of challenge, challenge type (*E. acervulina* vs. mix *Eimeria* spp.), and the dose response to *E. acervulina.* Surprisingly, the challenge and challenge type interaction effects were only significant for Ile, Leu, and Val, where the difference in digestibility values between nonchallenged and challenged birds was more affected with the *E. acervulina* challenge compared to the mixed *Eimeria* challenge. When looking at the dose response model, branched-chain AA (**BCAA**) digestibility showed both linear and quadratic reduction to *E. acervulina* oocyst concentration. Branched chain AA such as Leu, Ile, and Val, are essential AA for protein synthesis (Holeček, 2018). Contrary to most AA, BCAA catabolism mainly occurs in the skeletal muscle rather than the liver due to the high activity of BCAA aminotransferase (Holeček, 2018). This allows BCAA to be readily available to provide the α-amino group for endogenous synthesis of Gln and Ala, both of which are major energy substrates for cells of the immune system (Li et al., 2007; Holeček, 2018). There are a number of studies that reported immune impairment as a result of inadequate BCAA intake (Li et al., 2007). In a study by Konashi et al. (2000), BCAA deficiency significantly decreased relative thymus and bursa weights in 24-day-old broiler chickens, supporting a similar observation seen in Aschkenasy (1975) in Ile and Val-deficient diets fed to rats. The total hemagglutinin titres against sheep erythrocytes was also shown to be lower in birds fed BCAA-deficient diets (Konashi et al., 2000). Similarly, Bhargava et al. (1970) observed reduced antibody production in Newcastle-infected broiler chicks fed Val-deficient diets (Konashi et al., 2000). In another study by Tsukishiro et al. (2000), an increase in liver-associated lymphocytes and natural killer (**NK**) cells was seen in tetrachloride-induced cirrhotic rats fed a 14% casein diet supplemented with 10% BCAA compared to tetrachloride-induced cirrhotic rats fed a 24% casein diet. While many aspects of BCAA and its effects on the immune system are still unknown, it is speculated that BCAA have the greatest potential to modulate immune responses as they are essential for the function and protein synthesis of immune cells (Calder, 2006). Although it is unclear why BCAA digestibility seemed to be more impacted by *E. acervulina* than the mixed spp. challenge, perhaps it is the functional role of BCAA plays in the immune system in combination with the fact that *E. acervulina* commonly affects the duodenum and proximal jejunum, the major sites for AA digestion and absorption, that may provide a reason for the interaction effect on these AA in particular.

The challenge effect was shown to significantly influence AID of AA, except Trp, such that the challenged birds had lower ileal digestibility compared to the nonchallenged birds. It is hypothesized that the increased mucogenesis, rapid turnover of intestinal cells, and plasma protein leakage inflicted by the damage caused from the *Eimeria* life cycle contribute to the increase in endogenous AA loss (Adedokun et al., 2012; Adedokun and Adeola, 2016; Kim et al., 2022; Teng et al., 2021). Although apparent digestibility values are not corrected for endogenous loss, which may underestimate actual digestibility values in *Eimeria*-infected broilers, the values for AID are a representation of the net disappearance from the digestive tract and prior to the distal ileum, thus providing insight on the impact of an *Eimeria* challenge on AA digestibility as a whole (Stein et al., 2007). Even though Trp digestibility was not significantly affected by overall challenge, Trp values were lower in the mixed spp. challenge compared to the *E. acervulina* challenge and decreased with the mixed spp. challenge. Additionally, Trp was the only AA in which there was no linear or quadratic response to *E. acervulina* oocyst concentration. This was surprising as acute phase proteins such as fibrinogen, C-reactive protein, and haptoglobin, all contain a high concentration of Trp, therefore, the synthesis of acute phase proteins during an inflammatory response, as with an *Eimeria* infection, may have a higher requirement for Trp (Le Floc'h et al., 2011). Furthermore, intracellular apicomplexan parasites, such as *Toxoplasma gondii and Eimeria* spp., can induce an interferon (**IFN**)-γ -driven activation of host indoleamine 2,3-dioxgyenase (**IDO**), the rate-limiting enzyme of Trp catabolism in the kynurenine pathway (Schmid et al., 2012). Perhaps the infection caused by *E. acervulina* alone was not enough to elicit an effect on Trp digestibility but rather a combined effect of multiple *Eimeria* spp. However, when looking at the challenge type effect on AID, with exception to Ile, Leu, and Trp, the other indispensable AA were more severely affected by *E. acervulina* challenge than the mixed *Eimeria* spp. challenge. In a study by Su et al. (2014), the expression of aminopeptidase N and several AA transporters was shown to be downregulated in the duodenum of *E. acervulina-*infected broilers. As both components of the brush border membrane play a crucial role in efficient digestion of protein and uptake of AA, indeed the downregulation of these genes would result in reduced influx of essential AA (Paris and Wong, 2013; Su et al. 2014). Additionally, the studies that used an *E. acervulina* challenge had a higher concentration of oocysts in the dose than the studies that had used a mixed spp. challenge. As shown from our dose response models, all AA except Trp had a linear response to oocyst concentration in the dose while most AA except Cys, Pro, Glu, Arg, Met, and Trp demonstrated a quadratic effect. However, the lower concentration of oocysts in the mixed spp. challenge may potentially be due to the potency of using various *Eimeria* spp. instead of just a single spp.

When we compared the model slopes of challenge using 2-sample *t* tests, Cys was ranked most affected, with Arg, Met, Ala, Tyr, and Thr thereafter. Chemokines, a specialized type of cytokine used to induce migration of white blood cells to infected or damaged tissues, are made up of highly conserved cysteine residues (Miller and Mayo, 2017). Before chemokines bind to their respective receptors to stimulate chemotaxis, the conserved cysteine residues pair up to form disulfide bridges in order to maintain structural integrity (Miller and Mayo, 2017). However, the formation of disulphide cross-linking can restrict proteolytic enzyme attack, thus making cystine less digestible than cysteine (Ljøkjel et al., 2004; Baker, 2005). It is widely known that T-cell inflammatory responses are the major immunological reactions that occur in chickens infected with *Eimeria* to protect against re-infection (Hong et al., 2006). In a study by Hong et al. (2006), expression levels of both proinflammatory cytokines and chemokines were upregulated following primary *E. acervulina* infections. This was also observed in Laurent et al. (2001), where certain inflammatory chemokines were strongly up regulated following an *E. tenella* infection. Additionally, sulfur-containing AA such as Cys and Met, are considered endogenous antioxidants due to their ability to defend cells against oxidative stress (Yang and Liao, 2019). While all AA are susceptible to oxidation, Met and Cys are the most susceptible to reactive oxygen species and by association protect other proteins from oxidative damage (Yang and Liao, 2019).

Arg is an essential AA for broilers and the substrate for the biosynthesis of nitric oxide (**NO**) (Allen, 1999). Production of NO has been shown to be an important mediator of innate and acquired immunity, as well as a toxic defense molecule against infectious and invading organisms (Allen, 1999). Reactive oxygen species (**ROS**) and NO are products from activated macrophages and other phagocytic leukocytes during an inflammatory response (Allen and Fetterer, 2002). At high concentrations, the potential for NO to react with hydrogen peroxide to form peroxynitrite increases (Li et al., 2007). Peroxynitrite will then oxidize proteins, AA, DNA, lipids, leading to cell injury and/or death (Li et al., 2007). Therefore, Arg is an important modulator for the immunohomeostasis of the mucosa (Yang and Liao, 2019).

Finally, given the mucogenic nature of coccidiosis and the critical role Thr has in maintaining the intestinal mucosal layer, it is not surprising to see that Thr digestibility is one of the AA most affected by *Eimeria* (Rochell et al., 2016a)*.* A thick mucus layer covers the gastrointestinal epithelium to serve as a protective barrier and the first line of defense against microbes in the luminal space (Cornick et al., 2015). Bacteria adhere to the oligosaccharide side chains of mucins, therefore preventing them from making contact with the epithelium (Cornick et al., 2015). While feeding dietary Thr above optimal growth requirements in support of intestinal health and performance during an *Eimeria* infection has produced varying results (Kidd et al., 2003; Star et al., 2012; Wils-Plotz et al., 2013), it is evident mucin dynamics may be sensitive to a Thr deficiency due to its high concentration and structural importance in the mucin protein (Horn et al., 2009). In addition, Thr is also an important structural component of immunoglobulin and may contribute to lymphocyte proliferation and activation (Zhang et al., 2016). A study by Wang et al. (2006) found that dietary supplementation of Thr increased antibody production, serum IgG levels, and jejunal mucosal IgG and IgA concentrations in young pigs challenged with *Escherichia coli*, supporting the notion that the immune system is responsive to dietary Thr intake. Given the large involvement of these AA and the high priority and partitioning of nutrients to develop the necessary immune response against *Eimeria,* this may be why the digestibility of Cys, Met, Arg, and Thr were most affected.

In conclusion, this study revealed that an *Eimeria* challenge does indeed decrease AA digestibility and that the challenge type may also influence the digestibility of certain AA. From our models, we created a hierarchy of the most affected AA to the least. According to the ranking, Cys, Arg, Met, Ala, Tyr, and Thr seem to be the most impacted by *Eimeria* in broilers, thus suggesting that these AA may be potentially limiting for broilers subjected to an *Eimeria* infection. Therefore, nutritional intervention of these AA during *Eimeria* infections or vaccination programs may be a key in mitigating the negative effects associated with an *Eimeria* challenge.
